# MiR-582-5p/miR-590-5p targeted CREB1/CREB5–NF-κB signaling and caused opioid-induced immunosuppression in human monocytes

**DOI:** 10.1038/tp.2016.4

**Published:** 2016-03-15

**Authors:** X Long, Y Li, S Qiu, J Liu, L He, Y Peng

**Affiliations:** 1 Department of Neurology, Sun Yat-Sen Memorial Hospital, Sun Yat-Sen University, Guangzhou, China; 2 Discipline of Physiology, School of Medical Sciences, the University of Adelaide, Adelaide, SA, Australia

## Abstract

Chronic opioid abusers are more susceptible to bacterial and viral infections, but the molecular mechanism underlying opioid-induced immunosuppression is unknown. MicroRNAs (miRNAs) are emerging as key players in the control of biological processes, and may participate in immune regulation. In this study, we investigated the molecular mechanisms in opioid-induced and miRNA-mediated immunosuppression, in the context of miRNA dysregulation in opioid abusers. Blood samples of heroin abusers were collected and analyzed using miRNA microarray analysis and quantitative PCR validation. The purified primary human monocytes were cultured *in vitro* to explore the underlying mechanism. We found that morphine and its derivative heroin significantly decreased the expression levels of miR-582-5p and miR-590-5p in monocytes. cAMP response element-binding protein 1 (CREB1) and CREB5 were detected as direct target genes of miR-582-5p and miR-590-5p, respectively, by using dual-luciferase assay and western bolt. Functional studies showed that knockdown of CREB1/CREB5 increased tumor necrosis factor alpha (TNF-α) level and enhanced expression of phospho–NF-κB p65 and NF-κB p65. Our results demonstrated that miR-582-5p and miR-590-5p play important roles in opioid-induced immunosuppression in monocytes by targeting CREB1/CREB5–NF-κB signaling pathway.

## Introduction

Chronic therapeutic or addictive opioid use has been associated with susceptibility to bacterial and viral infections.^1^ Morphine, a commonly administered and studied opioid, is able to influence a variety of immune functions. For example, chronic morphine use is associated with increased expression of cytokines and chemokines, the signaling molecules of the innate immune system.^1, 2, 3^ Thus, the immune dysregulatory actions of morphine may be due to alterations to the innate immune system, which is the body's first line of defense against pathogens. The innate immune system helps to control and eliminate infections at an early stage by activating pro-inflammatory signaling cascades through secretion of cytokines and co-stimulatory molecules that trigger the inflammatory process.^4^ Alternations to cytokine and chemokine production would therefore influence the body's inflammatory response. The immune actions of morphine are commonly investigated using peripheral blood mononuclear cells (PBMCs), which include monocytes, lymphocytes and dendritic cells. Previous studies have found a correlation between morphine withdrawal and PBMC gene expression patterns, therefore suggesting that PBMCs are altered in morphine addiction models.^5, 6^ Together with the central role of monocytes in the innate immune system, PBMCs are promising targets for investigating the innate immune mechanisms of addiction.

MicroRNAs (miRNA) are emerging as key players in the control of biological processes, and the stage-specific expression of certain miRNA in the immune system suggests that they may participate in immune regulation.^7^ Binding of the miRNA guide strand to the miRNA recognition element within the 3′ untranslated region (3′ UTR) of target mRNA leads to repression of translation and eventually to mRNA destabilization and degradation.^8^ MiRNA may impact physiological processes by regulating the concentrations of just a few key cellular proteins that may be components of a single or of functionally interrelated pathways in a given cellular context.^9^ MiRNAs have been recently investigated during the effect of morphine on the immune system, and our previous study showed that morphine suppressed the innate immunity in microglia and bone marrow-derived macrophages through differential regulation of toll-like receptors (TLRs). Moreover, chronic morphine treatment induced miRNA-124 in these cells and subsequently promotes immunosuppression by directly targeting a subunit of nuclear factor-kappa B (NF-κB) and tumor necrosis factor receptor-associated factor 6 (TRAF6).^10^ Li *et al.*^11^ also found that miRNA-873 inhibited morphine-induced macrophage apoptosis by elevating A20 expression. However, few studies reported the effects of miRNAs in morphine-induced immune changes in human immune cells.

In this study, through miRNA microarray analysis, we found that the levels of miRNA-582-5p and miRNA-590-5p in PBMCs of heroin abusers were decreased significantly as compared with those in healthy controls. We further utilized purified primary human monocytes to investigate the potential involvement of miR-582-5p and miR-590-5p in opioid-induced immunosuppression. Finally, we demonstrated that miR-582-5p and miR-590-5p play important roles in opioid-induced immunosuppression in monocytes by targeting cAMP response element-binding protein 1/5 (CREB1/CREB5) NF-κB signaling pathway.

## Materials and methods

### Patients

Thirty-three heroin abusers (30 men/3 women) and 20 healthy donors (18 men/2 women) in the same age range participated in this study after giving informed consent. Heroin abusers were patients in Guangzhou Psychiatric Hospital in the People's Republic of China before detoxification treatment. They were identified as HIV antibody negative and without active infections, inflammatory diseases or chronic systemic illness. Further drug tests were performed on urine samples of the patients, and those who exhibited other substance abuse were excluded. Healthy donors with no history of drug or alcohol abuse or major medical disorders were recruited using convenience sampling from the community in which the study site was located. The institutional ethics review board of Sun Yat-Sen Memorial Hospital granted approval for the study. Informed consents were obtained from all involved subjects.

### *In vitro* morphine assay

Urine samples of heroin abusers were collected at day 1 of hospitalization. Morphine was detected by using test strip immunoassay for morphine (Guangzhou Wondfo Biotech, Guangzhou, China). In brief, urine sample was added to the sample well of the test strip and incubated for 2 min at room temperature. Invisible signal of test line and visible signal of reference line were decided as positive for morphine.

Human peripheral monocytes *in vitro* were treated with morphine sulfate (Northeast Pharmaceutical Group, Shenyang, China) at concentrations of 10^−2^, 10^−10^, 10^−8^, 10^−6^  m for 0, 4, 8, 12, 24 h.

### Cell separation, purification and culture

PBMCs were isolated from 10 ml heparin-treated blood of heroin abuser and healthy donors by density gradient centrifugation on Ficoll-Paque Plus (GE Healthcare, Buckinghamshire, UK). The cells were centrifuged and the plasma collected and frozen at −80 °C. After two washes in phosphate-buffered saline (PBS) and over a Percoll gradient, the monocyte-enriched fraction (~84.70%) was obtained. The percentage of monocytes was evaluated by flow cytometry using CD14 as a detection marker.

Monocytes were purified (~97.72%) with MACS Human Monocyte Isolation Kit II (Miltenyi Biotec, Bergisch Gladbach, Germany) from buffy coats of healthy donors obtained from GuangZhou Blood Center after centrifugation according to the above methods. Monocytes (5 × 10^6^ ml^−1^) then were resuspended in RPMI medium 1640 (GIBCO, Grand Island, NY, USA) supplemented with 10% fetal bovine serum (GIBCO), plated and treated as described. The 293T cell lines were grown in DMEM (Hyclone, Logan, UT, USA) supplemented with 10% fetal bovine serum.

### RNA extraction and miRNA microarray

Total RNA was isolated using TRIzol (Invitrogen, Carlsbad, CA, USA) and miRNeasy mini kit (QIAGEN, Hilden, Germany) according to manufacturer's instructions. RNA quality and quantity were measured by using NanoDrop SmiRCURYpectrophotometer (ND-1000, Nanodrop Technologies, Wilmington, DE, USA) and RNA integrity was determined by gel electrophoresis.

RNA extracted from the samples were labeled using the miRCURY Hy3/Hy5 Power labeling kit (Exiqon, Vedbaek, Denmark) according to the manufacturer's guideline and then hybridized on the miRCURY LNA Array (Version 18.0) (Exiqon) according to array manual. Differentially expressed miRNAs were identified through Fold Change filtering. Hierarchical clustering was performed using MEV software (v4.6, TIGR, Boston, MA, USA).

### RT–PCR and qPCR

Reverse transcription (RT) of the total RNA and real-time PCR were performed using All-in-One miRNAqRT–PCR Detection Kit (GeneCopoeia, Rockville, MD, USA) according to the manufacturer's protocol. All real-time PCR amplifications were performed in duplicate, using SYBRGreen technology of a 7500 real-time PCR System (Applied Biosystems, Foster City, CA, USA). All samples were calculated by the 2^−ΔΔCt^ method for relative quantification and the data were normalized to internal control U6 small nuclear RNA as previously reported.^12^ Specific primers and probes were obtained from Invitrogen. The primer sequences are listed in Supplementary Table S1.

### Transient miRNA/siRNA transfection

Freshly purified monocytes (5 × 10^6^ ml^−1^) were seeded on 12-well plates, transfected with miRNA mimics at a final concentration of 50 nm or transfected with miRNA inhibitor at a final concentration of 100 nm using Lipofectamine 2000 reagent (Invitrogen), according to the manufacturer's instructions. Synthetic hsa-miR-582-5p mimics, hsa-miR-582-5p inhibitor, hsa-miR-590-5p mimics, hsa-miR-590-5p inhibitor and the Allstars Negative Control small interfering RNA (siRNA) were purchased from Gene Pharma (Suzhou, China). CREB1 and CREB5 siRNA were also purchased from Gene Pharma. The miRNA and siRNA sequences are given in Supplementary Table S2.

### Cytokine response

Cytokine concentrations of tumor necrosis factor alpha (TNF-α) and interleukin-10 (IL-10) in cell-free plasma and supernatants were measured by the BD CBA Human Soluble Protein Flex Set system (Becton Dickinson, Franklin Lakes, NJ, USA). This system uses the sensitivity of amplified fluorescence detection with flow cytometry to measure a soluble analyte. All samples were acquired and analyzed on a FACSVerse (Becton Dickinson).

### Plasmid construction and luciferase reporter assay

CREB1/CREB5 mRNA 3′ UTRs containing the miR-582/miR-590-binding sequences for the human CREB1/CREB5 gene were amplified by means of PCR with human genomic DNA as a template. The PCR product was inserted into the Psichek-2 vector (Promega, Madison, WI, USA), and insertion was confirmed by means of sequencing. Binding-region mutations were achieved using a QuikChange Site-Directed Mutagenesis Kit (Stratagene, La Jolla, CA, USA) following the manufacturer's instructions.

For the luciferase reporter assays, transient transfection of 293T cells was carried out in 12-well plates with Lipofectamine 2000, according to the manufacturer's instructions. The cells were co-transfected with the luciferase constructs and each miRNA or scramble control RNA. One day after transfection, the cells were assayed with a luciferase assay kit (Promega) according to the manufacturer's instructions.

### Western blot analysis

The cells were washed with PBS twice and lysed using lysis buffer on ice for 30 min. Protein fractions were collected by centrifugation at 14 000 r.p.m. at 4 °C for 15 min and then subjected to SDS–PAGE and transferred to polyvinylidenedifluoride membranes. The membranes were blocked with 5% BSA and incubated overnight at 4 °C in primary antibody CREB5 (1:1000, Sigma, St Louis, MO, USA), CREB1 (1:1000, Cell Signaling Technology, Beverly, MA, USA), p–CREB (1:1000, Cell Signaling Technology), p65 (1:1000, Cell Signaling Technology), p–p65 (1:1000, Cell Signaling Technology) and glyceraldehyde 3-phosphate dehydrogenase (GAPDH, 1:10 000, Bioworld Technology, St. Louis Park, MN, USA). The blots were washed three times with TBST buffer and then incubated for 1 h at room temperature with anti-rabbit or anti-mouse secondary antibody conjugated with horseradish peroxidase. Western blot analysis was visualized using an enhanced chemiluminescence kit (Pierce, Beverly, MA, USA).

### Statistical analysis

The data are expressed as the mean±s.d. Statistical analysis was performed with one-way analysis of variance followed by Student's *t*-test for comparison between groups. Coefficients of partial correlation between variables were calculated using Spearman's analysis. All statistical analyses were performed with SPSS software, version 13.0 (Chicago, IL, USA). The *P-*values<0.05 were considered as statistically significant.

## Results

### Differentially expressed and validated miRNAs in PBMC

**Figure 1:**
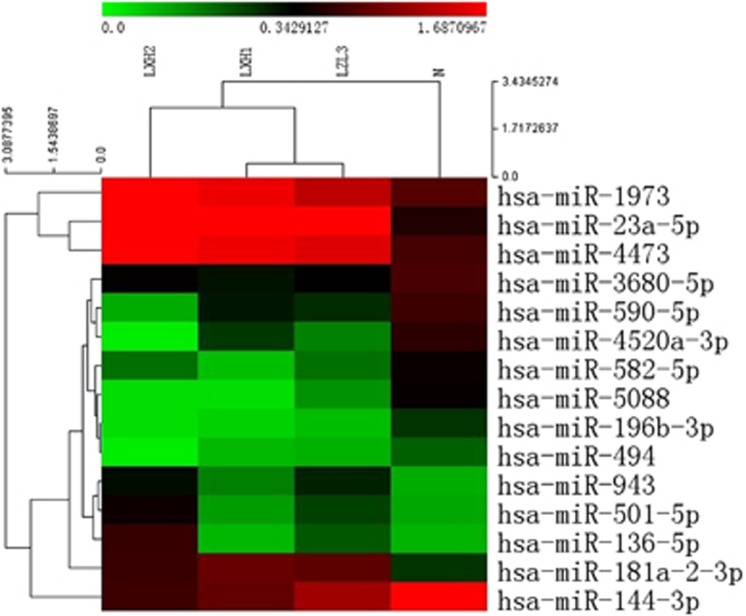
Heat map of 15 differentially expressed miRNAs in three heroin abusers (LXH2&LXH1&LZL3) compared with 1 healthy control (*N*). The heat map is an intensity plot with relative upregulation indicated in red, and downregulation indicated in green. miRNA, microRNA.

**Figure 2:**
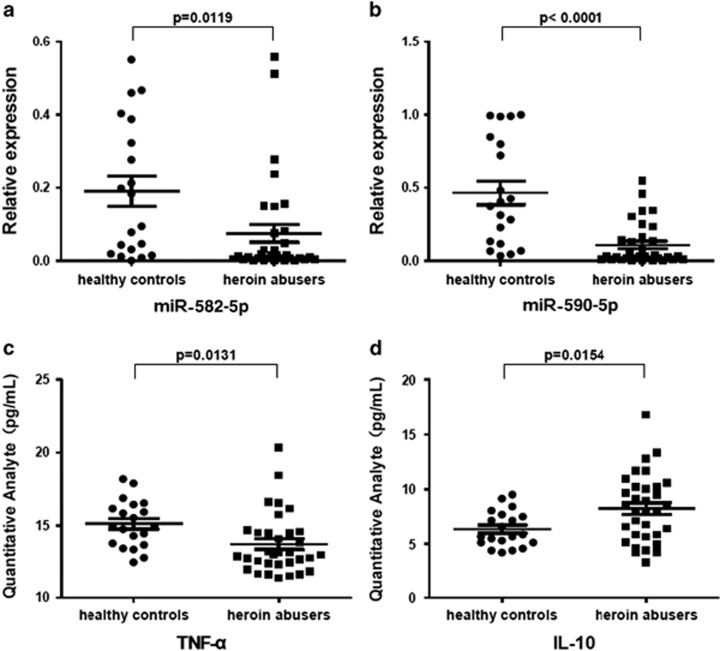
Validation of deregulated miRNAs by qPCR. An independent validation cohort included 33 heroin abusers and 20 healthy controls. MicroRNA abundance was normalized to U6 RNA. Quantification was presented as mean values (error bars corresponded to s.d.) relative to control from three independent experiments. (**a** and **b**) qPCR for various miRNAs showed downregulation of miR-582-5p/miR-590-5p in PBMC from 33 heroin abusers and 20 healthy controls. (**c** and **d**) Plasma concentrations of TNF-α and IL-10 in 33 heroin abusers compared with 20 healthy controls. miRNA, microRNA; qPCR, quantitative real-time PCR.

We collected the peripheral vein blood samples of 33 heroin abusers and 20 healthy controls between January 2013 and October 2013. The demographic data were summarized in Table 1. The blood samples of 3 heroin abusers and 1 healthy control were randomly selected and a total of 1904 miRNAs in PBMC were detected by using miRNA microarray. We identified 15 differentially expressed miRNAs in heroin abusers as compared with the healthy control (Figure 1). Furthermore, we detected these 15 miRNAs via quantitative real-time PCR (qPCR) analysis in 33 heroin users and 20 healthy controls (Table 1). We found a statistically significant downregulation of miR-582-5p (Figure 2a) and miR-590-5p (Figure 2b) in heroin abusers, as compared with healthy controls.

### Correlations between miRNAs and inflammatory cytokines

**Figure 3:**
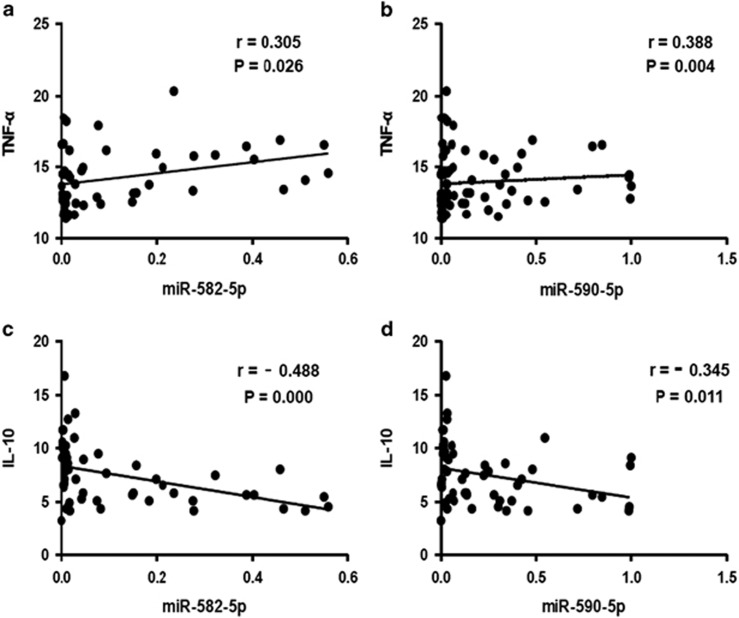
Correlations between miRNAs and inflammatory cytokines. Scatter plots to show the correlation between miR-582-5p and TNF-α (**a**), miR-582-5p and IL-10 (**b**), miR-590-5p and TNF-α (**c**) and miR-590-5p and IL-10 (**d**). miRNA, microRNA.

As shown in Figure 2, the plasma levels of TNF-α in heroin abusers were significantly decreased as compared with those in healthy controls. The plasma levels of IL-10 in heroin abusers were significantly increased as compared with those in healthy controls. As shown in Figure 3, there was a positive correlation between TNF-α and miR-582-5p/miR-590-5p (*P*=0.026 and *P*=0.004, respectively). There was a negative correlation between IL-10 and miR-582-5p/miR-590-5p (*P*=0.000 and *P*=0.011, respectively).

### MiR-582-5p/miR-590-5p and NF-κB–p65 expression in morphine-stimulated monocytes

**Figure 4:**
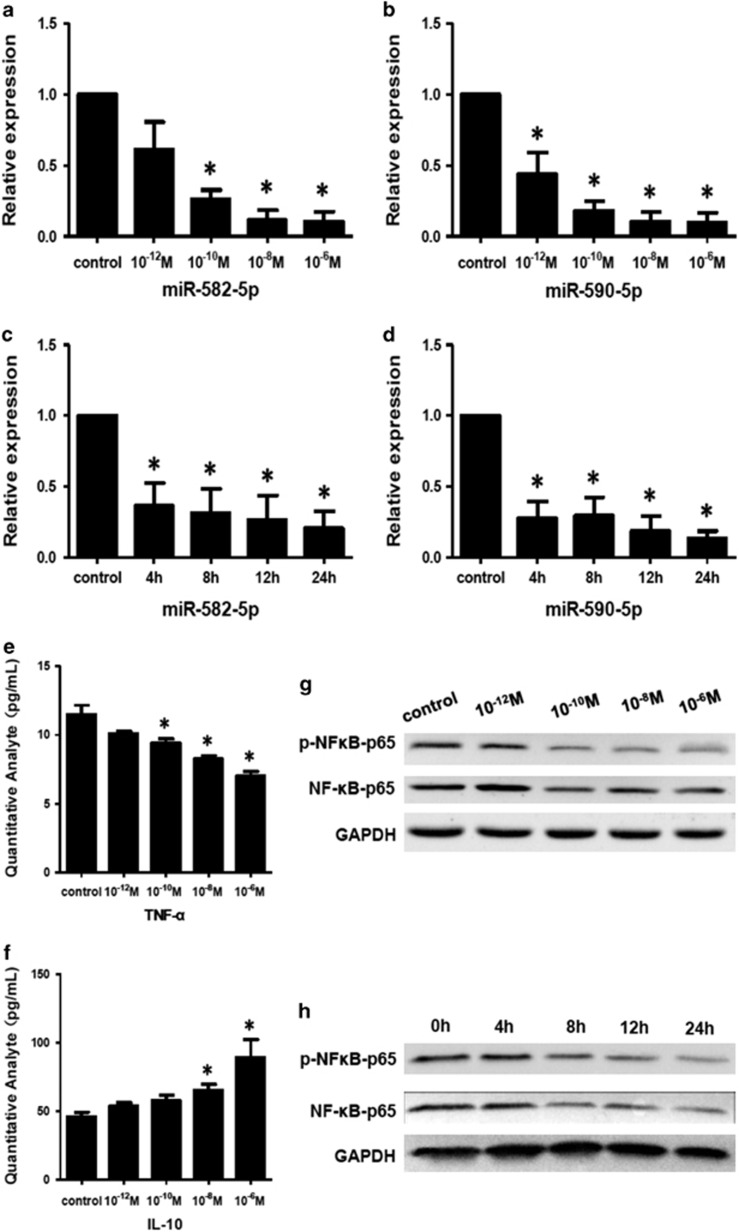
Time- and dose-dependent effects of morphine on miRNA, cytokine and NF-κB expression. (**a** and **b**) Concentration-dependent downregulation of miR-582-5p and miR-590-5p observed after stimulation with morphine (10^-6^–10^−12^ m) for 24 h in freshly purified human monocytes, as determined by qPCR. (**c** and **d**) Time-dependent downregulation of miR-582-5p and miR-590-5p observed after stimulation with morphine (10^−6^ m) for 0–24 h in human freshly purified monocytes. The data are expressed as the means and s.d. of a triplicate qPCR and are representative of three independent experiments. *P<0.01, compared with the control group. (**e** and **f**) Supernatant concentrations of TNF-α and IL-10 in morphine-stimulated monocytes compared with the control group. (**g** and **h**) Western blot analysis showed significant downregulation of p–NF-κB–p65 and NF-κB–p65 in morphine-stimulated monocytes. miRNA, microRNA; qPCR, quantitative real-time PCR.

To further investigate the mechanism of immunosuppression induced by morphine and its derivations, we detected the expression of miR-582-5p and miR-590-5p in morphine-pretreated human peripheral monocytes *in vitro* using qRT–PCR. As compared with the control group, morphine stimulation significantly decreased the expression levels of miR-582-5p and miR-590-5p in human monocytes in a time- and dose-dependent manner (Figure 4). After 10^−6^ m morphine stimulation for 24 h, the levels of miR-582-5p and miR-590-5p in monocytes decreased by 0.23-fold and 0.18-fold, respectively (*P*<0.01).

As shown in Figures 4e and f, morphine stimulation in human monocytes significantly decreased the supernatant level of TNF-α, and increased the supernatant level of IL-10. Furthermore, as compared with the control group, morphine stimulation significantly decreased phospho–NF-κB p65 and NF-κB p65 in monocytes in a time- and concentration-dependent manner (Figures 4g and h).

### MiR-582-5p and miR-590-5p modulated inflammatory cytokine production and NF-κB–p65 in monocytes

**Figure 5:**
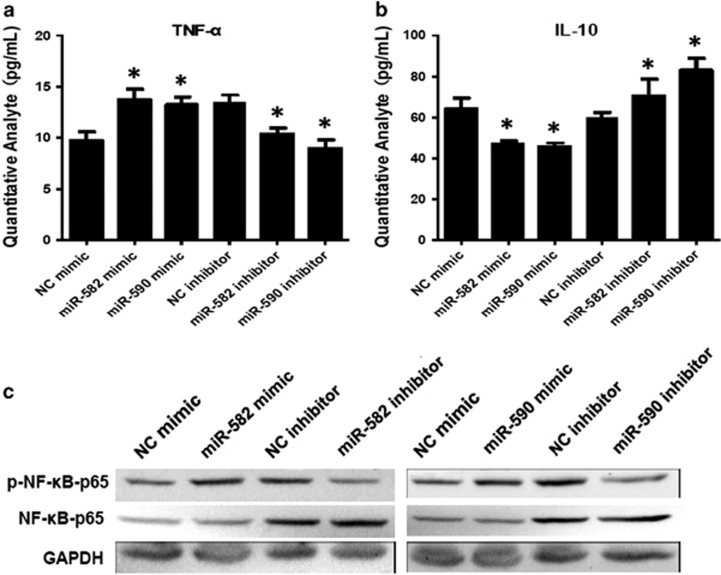
The effects of miR-582-5p and miR-590-5p on cytokine production. (**a** and **b**) MiR-582-5p and miR-590-5p transfection of human monocyte significantly increased supernatant TNF-α and decreased supernatant IL-10 levels following miR-582-5p and miR-590-5p transfection. Conversely, transfection with the miR-582-5p and miR-590-5p inhibitor significantly inhibited TNF-α production but increased IL-10 levels compared with control. Data expressed as the mean and s.d. of three independent experiments done in triplicates. *P<0.05, compared with the control group. (**c**) As determined by western blot analysis. Transfection with miR-582-5p/miR-590-5p mimics significantly increased the levels of phospho–NF-κB–p65 in monocyte. Conversely, transfection with the miR-582/miR-590 inhibitor significantly decreased the levels of phospho–NF-κB–p65 in monocytes. miRNA, microRNA.

We transfected the monocytes with miR-582-5p and miR-590-5p mimics reagents or inhibitor reagents and detected the levels of TNF-α and IL-10 in the supernatant. The levels of TNF-α significantly increased after miR-582-5p/miR-590-5p mimic transfection, and significantly decreased after miR-582-5p/miR-590-5p inhibitor transfection (Figure 5a). Moreover, IL-10 levels significantly decreased after miR-582-5p/miR-590-5p mimic transfection, and significantly increased after miR-582-5p/miR-590-5p inhibitor transfection (Figure 5b).

Furthermore, we found that the expression of phospho–NF-κB–p65 was significantly increased in the monocytes transfected with miR-582-5p/miR-590-5p mimics, and was significantly decreased in the monocytes transfected with miR-582-5p/miR-590-5p inhibitor (Figure 5c).

### MiR-582-5p/miR-590-5p targeted CREB1/CREB5 3′ UTR and caused translational suppression

**Figure 6:**
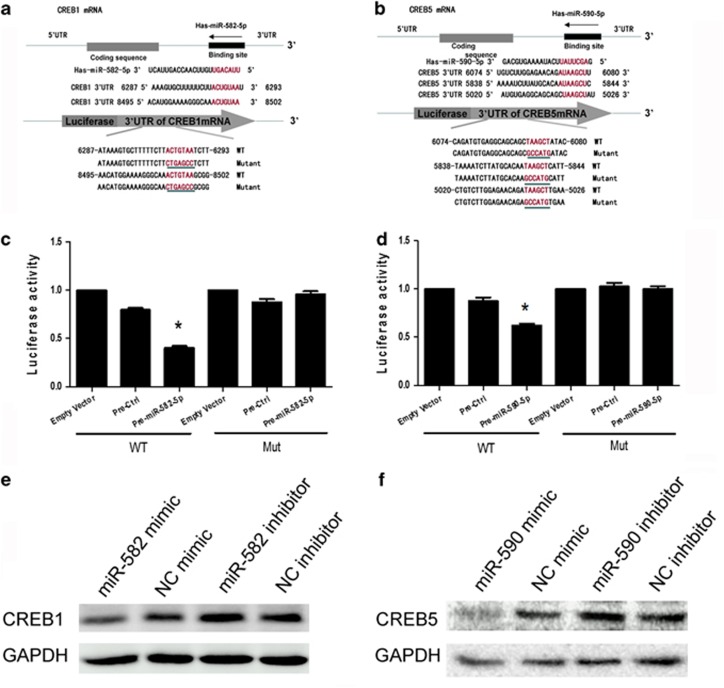
MiR-582-5p/miR-590-5p targets CREB1/CREB5 3′ UTR results in translational suppression. (**a**) CREB1 mRNA shows two potential binding sites in the 3′ UTR for miR-582-5p. (**b**) CREB5 mRNA shows three potential binding sites in the 3′ UTR for miR-590-5p. (**c** and **d**) Validation of binding of miR-582-5p/miR-590-5p with the 3′ UTR of CREB1/CREB5 by using luciferase reporter assays. Mut, Mutated CREB1/CREB5 3′ UTR; WT, wild-type CREB1/CREB5 3′ UTR. (**e** and **f**) CREB1/CREB5 expression in monocytes 24 h after transfection of either miR-582-5p/miR-590-5p precursor or anti- miR-582-5p/anti-miR-590-5p. Bars represent the mean±s.d. from three independent experiments. *P<0.05 versus control. miRNA, microRNA; UTR, untranslated region.

As shown in Figures 6a and b, we performed bioinformatics analysis with the three computer-aided algorithms TargetScan (Whitehead Institute, Cambridge, MA, USA), miRanda (Memorial Sloan-Kettering Cancer Center, New York, NY, USA) and PicTar (the Rajewsky lab at NYU's Center for Comparative Functional Genomics, New York, NY, USA, and the Max Delbruck Centrum, Berlin, Germany). These algorithms predicted that miR-582-5p/miR-590-5p could potentially target the sequence within the 3′ UTR of CREB1/CREB5 of various animal species, including humans. We characterized the binding of the miRNAs to the CREB1/CREB5 3′ UTR and the inverse correlation between the levels of miR-582-5p/miR-590-5p and CREB1/CREB5. 293T cells were transfected with a CREB1/CREB5-luciferase reporter construct, wherein luciferase expression was regulated by the CREB1/CREB5 3′ UTR, a miR-582-5p/miR-590-5p potential binding element. Co-transfection of 293T cells with both miR-582-5p/miR-590-5p with CREB1/CREB5-luciferase constructs resulted in a significant decrease in luciferase activity, suggesting the preferential binding of miR-582-5p/miR-590-5p with the 3′ UTR of CREB1/CREB5. As a negative control, 293T cells were also co-transfected with a construct containing mutations in the miR-582-5p/miR-590-5p-binding region of the 3′ UTR–CREB1/CREB5, which conversely did not result in reduced luciferase activity (Figures 6c and d).

To further investigate the role of miR-582-5p/miR-590-5p in regulating the CREB1/CREB5 chain translation, we transfected the human peripheral monocytes with the precursor miR-582-5p/miR-590-5p *in vitro* and subsequently examined the expression of CREB1/CREB5 protein. As shown in Figures 6e and d, monocyte transfected with the precursor miR-582-5p/miR-590-5p showed reduced expression of CREB1/CREB5 protein as compared with monocytes transfected with an unrelated precursor miRNA control. Transfection of anti- miR-582-5p/anti-miR-590-5p (to knock down endogenous miR-582-5p/miR-590-5p) resulted in enhanced expression of CREB1/CREB5. These results underpin the role of miR-582-5p/miR-590-5p in regulating CREB1/CREB5 protein expression via a post-transcriptional mechanism.

### MiR-582-5p/miR-590-5p targeted CREB1/CREB5 to promote NF-κB activity

**Figure 7:**
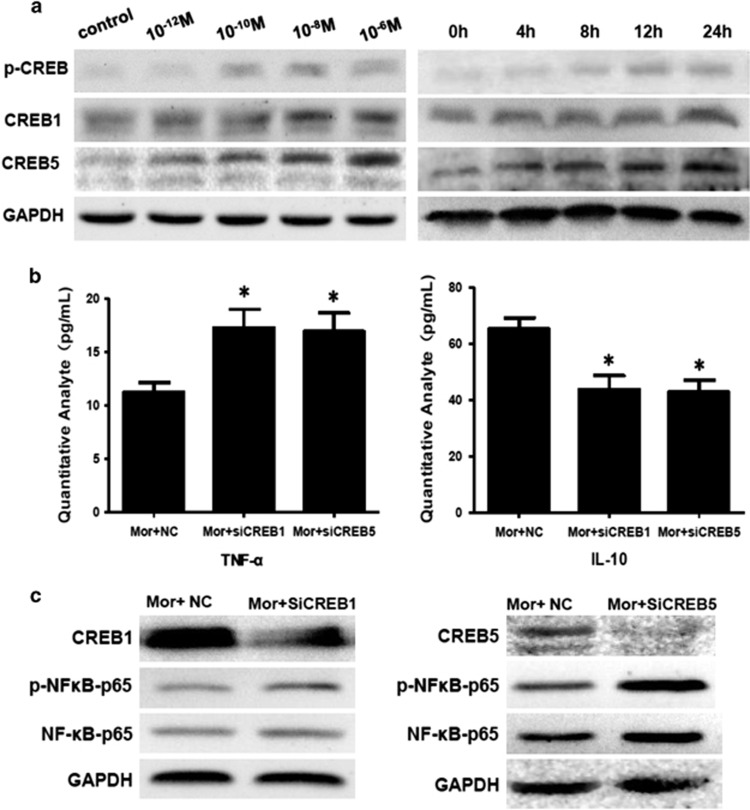
The effects of miR-582-5p/miR-590-5p on NF-κB signaling. (**a**) Morphine stimulation significantly increased the expression levels of phospho–CREB, CREB1 and CREB5 in monocytes in a time (0–24 h)- and concentration (10^−12^–10^−6^ m)-dependent manner in freshly purified human monocytes. (**b** and **c**) Morphine-stimulated monocyte transfected with siCREB1/siCREB5 demonstrated increased TNF-α levels, but reduced IL-10 levels and enhanced expression of phospho–NF-κB–p65 and NF-κB–p65 protein compared with untransfected morphine-stimulated monocytes.

Morphine stimulation significantly increased the expression levels of phospho–CREB, CREB1 and CREB5 in a time- and concentration-dependent manner, as compared with control (Figure 7a).

To investigate the underlying molecular mechanisms of this effect, we examined whether the NF-κB signaling pathways were involved in miR-582-5p/miR-590-5p-suppressed CREB1/CREB5. Morphine-stimulated monocytes were transfected with siRNAs of CREB1 and CREB5. As shown in Figures 7b and c, transfection of siCREB1/siCREB5 in morphine-stimulated monocytes resulted in significantly increased TNF-α levels and significantly decreased IL-10 levels in the supernatants, and enhanced expression of phospho–NF-κB–p65 and total NF-κB–p65 protein in the monocytes, as compared with morphine-stimulated monocytes without transfection (Figures 7b and c).

## Discussion

In the current study, we detected the expression of miRNAs in PBMCs from heroin abusers before detoxification and healthy controls. Microarray profiling identified 15 differentially expressed miRNAs. Only miR-582-5p and miR-590-5p were confirmed to be significantly reduced in heroin abusers by using qPCR analysis. Morphine stimulated the purified primary human monocytes *in vitro*, upregulated the protein level of CREB1/CREB5 and downregulated miRNAs miR-582/miR-590. We further demonstrated that CREB1/CREB5, as the targets of miR-582-5p/miR-590-5p, negatively regulated the activity of NF-κB. The present study therefore revealed that the downregulation of critical miRNAs may function as a molecular mechanism in opioids-induced immunosuppression.

In this study, miR-582-5p and miR-590-5p were decreased by morphine simultaneously and showed the same pro-inflammatory functions. This might suggest the similarity in their functional targets. Co-transfection of miR-582-5p/miR-590-5p using 3′ UTR luciferase reporters identified CREB1/CREB5 as their targets. CREB1 aliases CREB and CREB5 aliases CRE-BPA are all members of the CREB family that function as transcription factors. Although previous studies mainly focused on the effect of CREB on opioid dependence, for example, morphine condition place preference,^13^ emerging evidence has revealed the function of CREB in immune responses, including macrophage survival, regulation of T and B lymphocytes and inducing transcription of immune-related genes, for example, IL-10.^14^ CREB-mediated immune regulation could be via the inhibition of NF-κB activity. Wen *et al.*,^15^ hypothesized that a balance between CREB and CREB co-activator, CREB-binding protein (CBP)/p300 would determine whether the overall response leads to the inhibition or enhancement of NF-κB activity but the significance of this hypothesis is underdetermined. In our study, miR-582-5p- and miR-590-5p-transfected monocytes exhibited significantly increased levels of phospho–NF-κB. Furthermore, inhibition of CREB1 and CREB5 by transfection with siRNAs significantly promoted NF-κB phosphorylation in morphine-stimulated monocytes. Therefore, our findings highlight the role of CREB1 and CREB5 in the inhibition of the NF-κB signaling pathway in opioid-induced immunosuppression.

In our study, chronic heroin abuse *in vivo* and morphine treatment *in vitro* caused decreased TNF-α and increased IL-10 levels. TNF-α is an important mediator of progression of many immune diseases, due to its strong pro-inflammatory and immunostimulatory activities. As shown in our results as well as previous studies,^16, 17^ long-term heroin abuse resulted in a reduction in plasma expression of TNF-α as compared with health controls. It has been further demonstrated that TNF-α induction by morphine is NF-κB dependent.^18^ For full activation, NF-κB must be further modified by phosphorylation of its subunits and then translocated to the nucleus. In the nucleus, the p65 subunit of NF-κB has the opportunity to associate with the κB motif positioned at the TNF-α promoter and enhance its transcription.

IL-10, as a potent anti-inflammatory cytokine that limits inflammation and prevents unwanted tissue damage, is related to TNF-α,^19^ and negatively regulates TNF-α production in monocytes.^20^ The best-studied signaling for IL-10 regulation in monocytes is the TLR pathway.^21, 22^ Activation of TLRs results in the activation of several signaling pathways including mitogen-activated protein kinase (MARK) pathways, the phosphatidylinositol 3 kinase (PI3K)–Akt pathway, NF-κB pathway and the activation of interferon regulatory factors. These downstream pathways collectively regulate the production of TLR-induced cytokines, including IL-10. Since IL-10 transcription is promoted by the binding of phosphorylated CREB and activator protein 1 (AP-1) following MARKs activation, CREB plays an essential role in the production of IL-10. Conversely, IL-10, as an anti-inflammatory factor, can regulate the pathway for TLR-induced NF-κB activation through CREB. CREB and its co-activators, CBP/p300, can interact with NF-κB at the same region, and respectively cause decreased or enhanced NF-κB activity. IL-10 induces the production of glycogen synthase kinase-3 beta, which increases the binding of CREB, and decreases the binding of CBP/p300 to NF-κB.

MiRNAs have been implicated in innate and adaptive immunity. The regulation of 200 miRNAs following pro-inflammatory stimulation has been assessed by Taganov *et al.*^23^ and O'Connell *et al.*^24^ In this study, we found a positive correlation between the blood plasma levels of TNF-α and miR-582-5p/miR-590-5p in PBMCs, together with a negative correlation between the plasma level of IL-10 and miR-582-5p/miR-590-5p in PBMCs in heroin abusers. Further experiments *in vitro* showed increased TNF-α and decreased IL-10 level in miR-582-5p- and miR-590-5p-transfected monocytes. Our results indicate that miR-582-5p and miR-590-5p may be related to the innate immune response as pro-inflammatory factors. Although few studies have reported the effect of miR-582-5p/miR-590-5p on cytokines, it has recently been found that miR-590 alleviated pro-inflammatory cytokine secretion in human macrophages.^25^ In our study, miR-582-5p- and miR-590-5p-transfected monocytes exhibited significantly increased levels of phospho–NF-κB. Furthermore, inhibition of CREB1 and CREB5 proteins by transfection with siRNAs significantly promoted NF-κB phosphorylation in morphine-stimulated monocytes. Therefore, our findings highlight the role of miR-582-5p and miR-590-5p in the NF-κB signaling pathway in opioid-induced immune changes, and possess a two-step action in the production of cytokines during phases of miR-582-5p/miR-590-5p stimulation and inhibition.^26^ The effect of morphine on NF-κB likewise shows two-step action. For full activation, NF-κB must be further modified by phosphorylation of its subunits followed by translocation into the nucleus. Once in the nucleus, the p65 subunit of NF-κB has the opportunity to associate with the κB motif positioned at the target gene promoter to enhance its transcription. Pretreatment with a low dose of morphine (picomolar concentration) resulted in an increase in NF-κB activation. In contrast pretreatment with a high dose of morphine (micromolar concentration) led to a significant decrease in NF-κB activation.^1^ Our results thus suggest that morphine regulates NF-κB activation in a time- and concentration-dependent manner.

In PBMCs of heroin abusers, the levels of miR-582-5p and miR-590-5p were decreased significantly as compared with those in healthy controls. Further investigations using the purified primary human monocytes *in vitro* showed that miR-582-5p and miR-590-5p play important roles in opioid-induced immunosuppression in monocytes by targeting CREB1/CREB5–NF-κB signaling pathway.

## Figures and Tables

**Table 1:** Characteristics of heroin abusers and healthy controls

*Variable*^a^	*Healthy controls (*N=*20)*	*Heroin abusers (*N=*33)*	P*-value*
Age (year)	34 (24,50)	36 (22,53)	0.450
Male gender, *N* (%)	18 (90.0)	30 (90.9)	0.719
Heroin exposure time (mean)	—	12 years	

a Values are reported as median.
