# Supplementary material for: MiR-582-5p/miR-590-5p targeted CREB1/CREB5–NF-κB signaling and caused opioid-induced immunosuppression in human monocytes
Source: Transl Psychiatry. 2016 Mar 15;6(3):e757–. doi: 10.1038/tp.2016.4 (PMC4872460; doi:10.1038/tp.2016.4)
Supplement: Supplementary Information [file tp20164x1.doc]

Supplementary Table S1. PCR primers and oligonucleotide sequences

| Gene | Oligonucleotide Sequence(5’-3’) |
| --- | --- |
| hsa-miR-1973 | ACCGTGCAAAGGTAGCATA |
| hsa-miR-23a-5p | GGGGTTCCTGGGGATGGGATTT |
| hsa-miR-4473 | CTAGTGCTCTCCGTTACAAGTA |
| hsa-miR-3680-5p | GACUCACUCACAGGAUUGUGCA |
| hsa-miR-590-5p | GAGCTTATTCATAAAAGTGCAG |
| hsa-miR-4520a-3p | UUGGACAGAAAACACGCAGGAA |
| hsa-miR-582-5p | TTACAGTTGTTCAACCAGTTACT |
| hsa-mir-5088 | CCCAUCAGGGCUCAGGGAUUGGAUGGAGGUGAUGGGGGCAGGGGAUGGGUCUCACCCUCCCUUCUUCCUGGGCCCUCAG |
| hsa-miR-196b-3p | TCGACAGCACGACACTGCCTTC |
| hsa-miR-494 | TGAAACATACACGGGAAACCTC |
| hsa-miR-943 | CTGACTGTTGCCGTCCTCCAG |
| hsa-miR-501-5p | AATCCTTTGTCCCTGGGTGAGA |
| hsa-miR-136-5p | ACTCCATTTGTTTTGATGATGGA |
| hsa-miR-181a-2-3p | ACCACTGACCGTTGACTGTACC |
| hsa-miR-144-3p | TACAGTATAGATGATGTACT |
| U6 | CGCAAGGATGACACGCAAATTCGT |

Supplementary Table S2. miRNA and siRNA oligonucleotide sequences.

|  | Oligonucleotide Sequence(5’-3’) |
| --- | --- |
| hsa-miR-582-5p mimic | Sense UUACAGUUCAACCAGUUACU  Antisense UAACUGGUUGAACAACUGUAAUU |
| hsa-miR-590-5p mimic | Sense GAGCUUAUUCAUAAAAGUGCAG  Antisense GCACUUUUAUGAAUAAGCUCUU |
| Negative control mimic | Sense UUCUCCGAACGUGUCACGUTT  Antisense ACGUGACACGUUCGGAGAATT |
| Negative control inhibitor | 5’-CAGUACUUUUGUGUAGUACAA-3’ |
| SiRNA-CREB1 | Sense GCCACAGAUUGCCACAUUATT  Antisense UAAUGUGGCAAUCUGUGGCTT |
| SiRNA-CREB1 | Sense GACACAUGAUGGAGAUGAUTT  Antisense AUCAUCUCCAUCAUGUGUCTT |
|  | |
